# Treatment initiation for parkinson’s disease in Australia 2013–2018: a nation-wide study

**DOI:** 10.1186/s12877-022-03095-3

**Published:** 2022-06-03

**Authors:** Marjaana Koponen, J. Simon Bell, Samanta Lalic, Rosie Watson, Anne M. Koivisto, Jenni Ilomäki

**Affiliations:** 1 Centre for Medicine Use and Safety, Faculty of Pharmacy and Pharmaceutical Sciences, Monash University, Parkville, VIC Australia; 2 Kuopio Research Centre for Geriatric Care, University of Eastern Finland, Kuopio, Finland; 3 School of Pharmacy, University of Eastern Finland, Kuopio, Finland; 4 Department of Epidemiology and Preventive Medicine, Monash University, Parkville, VIC Australia; 5 Pharmacy Department, Monash Health, Melbourne, VIC Australia; 6 Population Health and Immunity Division, Walter and Eliza Hall Institute, Parkville, VIC Australia; 7 The Department of Medicine, Royal Melbourne Hospital, The University of Melbourne, Melbourne, VIC Australia; 8 Neurology, Institute of Clinical Medicine, University of Eastern Finland, Kuopio, Finland; 9 NeuroCenter, Neurology, Kuopio University Hospital, Kuopio, Finland; 10 Department of Neurosciences, University of Helsinki, Helsinki, Finland; 11 Geriatrics, Internal Medicine and Rehabilitation, Helsinki University Hospital, Helsinki, Finland; 12 Centre for Medicine Use and Safety, Monash University, 381 Royal Parade, Parkville, VIC 3052 Australia

**Keywords:** Antiparkinson drugs, Parkinson’s disease, Pharmacoepidemiology, Australia, Sex differences

## Abstract

**Background:**

Guidelines highlight the importance of an individualized approach to treatment initiation for Parkinson’s disease. Our aim was to investigate initiation of anti-Parkinson medication in Australia from 2013–2018, and to determine factors predicting choice of initial treatment.

**Methods:**

Cohort of new-users (*N* = 4,887) of anti-Parkinson medication aged ≥ 40 years were identified from a 10% random representative sample of national medication dispensing data from July-2013 to June-2018. Changes in treatment initiation were examined across the whole cohort and stratified by age and sex.

**Results:**

Treatment initiation was most frequent with levodopa followed by non-ergot dopamine agonists (DAs) and anticholinergics. Two thirds initiated with levodopa across the study period. Initiation with non-ergot DAs increased from 22 to 27% (rate ratio, RR 1.23, 95% confidence interval, CI 1.02–1.47) and initiation with anticholinergics decreased from 6.9% to 2.4% (RR 0.34, 95% CI 0.21–0.55) from 2013–2018. Among persons aged ≥ 65 years, one third of women and one fourth of men initiated on levodopa. Among women aged < 65 years, rates of treatment initiation with DAs (37%) and levodopa (37%) were similar in 2013/2014 but initiation with DA exceeded levodopa thereafter. Among men aged < 65 years, treatment initiation with levodopa (44%-49%) remained more frequent than initiation with DAs (29%-32%) throughout the study period.

**Conclusions:**

Treatment initiation with levodopa was most frequent among persons aged ≥ 65 years, consistent with current guidelines. Whilst the value of levodopa sparing strategies is unclear, treatment initiation with DA has become increasingly common relative to levodopa among women but not among men aged < 65 years.

**Supplementary Information:**

The online version contains supplementary material available at 10.1186/s12877-022-03095-3.

## Background

Uncertainties exist in relation to the optimal initial treatment for Parkinson’s disease (PD). Clinical guidelines recognize that levodopa provides the greatest benefit for motor symptoms [1–6]. However, long-term levodopa treatment is associated with motor fluctuations. Clinicians often prefer levodopa-sparing strategies (e.g. non-ergot dopamine agonists (DAs) or monoamine oxidase B (MAO-B) inhibitors) as initial therapy, especially among younger patients at higher risk of motor fluctuations and dyskinesia. Nevertheless, evidence suggests long-term motor complications and motor function are similar with initiation of levodopa or DAs [7–9]. DAs have higher risk of neuropsychiatric adverse events than levodopa [9].

Initial medication selection is individualized according to clinical characteristics (e.g. predominant symptoms, symptom severity, disability), comorbidities, concomitant medications, individual life circumstances and treatment preferences [1–6]. The United Kingdom NICE guidelines recommend levodopa in the early stage if motor symptoms impact quality of life; and DAs, levodopa or MAO-B inhibitors if motor symptoms do not affect quality of life [4]. Other guidelines recommend non-ergot DAs or MAO-B inhibitors for younger patients or patients with mild symptoms [1–3, 5, 10]. Levodopa is often recommended for older patients, those with more severe symptoms, or at higher risk of cognitive and psychiatric adverse events. Connolly and Lang [10] suggest three decision trees according to predominant symptoms i.e. for ‘tremor-dominant motor symptoms’, ‘predominant bradykinesia and impaired dexterity’, and ‘predominant postural instability and gait impairment’. Differences in guideline recommendations are likely to contribute to within and between country differences in treatment initiation for PD.

The objective of this study was to investigate changes in initiation of anti-Parkinson medication in Australia from 2013–2018, and to determine factors that predict choice of first anti-Parkinson medication.

## Methods

### Study design and setting

This retrospective cohort study utilized Australian Pharmaceutical Benefit Scheme (PBS) data for a random representative 10% sample of the Australian population [11]. The PBS random sample has been made available to research by Services Australia. The 10% random sample is derived from a ‘one in ten’ random sample of patients eligible to the Australia’s universal health care system Medicare, and dispensed medications via the PBS. The PBS subsidizes prescription medications for Australia’s 25 million citizens, permanent residents and foreign visitors from countries with reciprocal health care agreements. The data contain person-level records of all reimbursed medications dispensed from community pharmacies, private hospitals and public hospital outpatient and discharge dispensing in all states except New South Wales and Australian Capital Territory. Since July 2012, all under co-payment are captured. Data include quantity dispensed, PBS item code, dispensing date, sex, birth year, death year, and concessional status. PBS item codes are mapped to the medication name, strength and Anatomical Therapeutic Chemical (ATC) Classification codes [12].

### Study sample

**Fig. 1:**
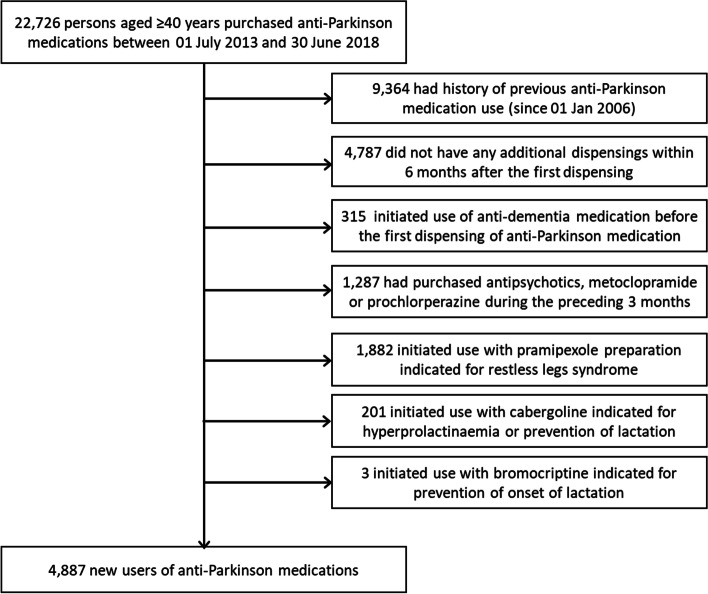
Formation of the study sample according to exclusion criteria

We first identified all persons aged ≥ 40 years first dispensed an anti-Parkinson medication (ATC code N04) between 1st July 2013 and 30th June 2018 (shown in Fig. 1). We excluded persons with previous anti-Parkinson medication use to identify incident users. We required initiators to have a second dispensing within 6-month from the first dispensing. We excluded persons with ≥ 1 dispensings of acetylcholinesterase inhibitors (N06DA) or memantine (N06DX01) prior to initiating an anti-Parkinson medication. In addition, we excluded persons with ≥ 1 dispensings of antipsychotics (N05A, excluding lithium), prochlorperazine (N05AB04) or metoclopramide (A03FA01) during the preceding three months. These exclusion criteria were applied because the diagnostic criteria for PD suggest that early dementia or onset of symptoms coinciding with dopamine antagonist treatment suggests diagnosis other than PD [13]. In Australia, two of the 12 available pramipexole products were only subsidized for persons with severe restless legs syndrome and persons initiating these two products were excluded. We also excluded persons who initiated cabergoline or bromocriptine products subsidized for hyperprolactinemia or prevention of the onset of lactation.

### Exposure

Persons dispensed a second anti-Parkinson medication within one month of their first anti-Parkinson medication (N04) were considered to have initiated on a combination regimen.

The initial anti-Parkinson medications were categorized into eight mutually exclusive groups:

- levodopa with peripheral decarboxylase inhibitor (N04BA02);
- anticholinergics (N04AA01 benzhexol, N04AA02 biperiden, N04AC01 benzatropine);
- non-ergot DAs (pramipexole N04BC05, apomorphine N04BC07, rotigotine N04BC09);
- ergot DAs (bromocriptine N04BC01, pergolide N04BC02, cabergoline N04BC06);
- MAO-B inhibitors (selegiline N04BD01, rasagiline N04BD02);
- amantadine (N04BB01);
- levodopa with entacapone (N04BA03 or N04BA02 + N04BX02) or entacapone only (N04BX02); and
- combination regimens of ≥ 2 different anti-Parkinson drug groups.

### Covariates

Concessional status (concessional or general) was defined based on initial anti-Parkinson dispensing. All other covariates were measured at baseline with a 12-month look-back period. Comorbidities were identified using medication dispensings as proxies for medical conditions using the Rx-Risk Index [14]. Comorbidities included cardiovascular disorders, diabetes, gastric acid disorder, reactive airway disease, and osteoporosis/Paget’s. As neuropsychiatric disturbances are a prediagnostic feature of PD [15], we considered antidepressants, benzodiazepines and related drugs, and antipsychotics as predictors. As antipsychotic users during preceding 3 months were excluded, use of antipsychotics was measured from 3 to 12 months prior baseline. Use of propranolol was included as a marker of tremor-dominant symptoms [10]. Use of opioids, nonsteroidal anti-inflammatory agents (NSAIDs), paracetamol and pregabalin were included as markers of pain because different types of pain are common in PD [16] (Supplementary Table 1).

### Statistical analyses

Characteristics of the new users were described as frequencies and percentages, or medians and interquartile ranges (IQR). Changes in treatment initiation were studied for the whole study sample and stratified by age group and sex. Poisson regression was used to calculate rate ratios (RRs) with 95% Confidence Intervals (CIs) for the initiation of different anti-Parkinson medications. The July 2013-June 2014 financial year, initiators aged ≥ 65 years, and men were used as reference groups when comparing the incidence rates between years, age groups and sexes, respectively. The associations between predictors and initial anti-Parkinson medication were analyzed with logistic regression and initiation of levodopa was used as the reference category. The odds ratios (ORs) with 95% CIs were adjusted for financial year and all the predictors included into the model. All analyses were conducted using SAS Version 9.4 (SAS Institute Inc., Cary, NC, USA).

## Results

The study sample comprised 4,887 new users of anti-Parkinson medication (shown in Fig. 1). The median age at initiation was 73 (IQR 63–80) years, 56% were men and 74% were concessional beneficiaries (Table 1). Three quarters were dispensed cardiovascular medications, 59% analgesics, 50% psychotropics, and 45% medications for gastric acid disorders during the preceding year.

**Table 1:** Characteristics of the new users (*n* = 4,887) of anti-Parkinson medications

	**Levodopa** (*n* = 3084)	**Non-ergot DAs** (*n* = 1184)	**MAO-B inhibitors** (*n* = 171)	**Anticholinergics** (*n* = 168)	**Combination of ≥ 2 groups** (*n* = 191)	**Total** (*n* = 4887)^a^
	n (%)	n (%)	n (%)	n (%)	n (%)	n (%)
Aged ≥ 65 years	2540 (82.4)	662 (55.9)	110 (64.3)	79 (47.0)	126 (66.0)	3553 (72.7)
Men	1839 (59.6)	537 (45.4)	114 (66.7)	91 (54.2)	112 (58.6)	2728 (55.8)
Concessional beneficiary	2395 (77.7)	825 (69.7)	98 (57.3)	124 (73.8)	127 (66.5)	3622 (74.1)
Use of propranolol	169 (5.5)	33 (2.8)	17 (9.9)	17 (10.1)	12 (6.3)	251 (5.1)
Use of any analgesic	1726 (56.0)	825 (69.7)	71 (41.5)	101 (60.1)	101 (52.9)	2873 (58.8)
Opioids	983 (31.9)	586 (49.5)	34 (19.9)	66 (39.3)	49 (25.7)	1746 (35.7)
NSAIDs	679 (22.0)	372 (31.4)	32 (18.7)	42 (25.0)	44 (23.0)	1191 (24.4)
Paracetamol	886 (28.7)	333 (28.1)	25 (14.6)	47 (28.0)	43 (22.5)	1351 (27.6)
Pregabalin	295 (9.6)	218 (18.4)	7 (4.1)	23 (13.7)	18 (9.4)	579 (11.8)
Use of any psychotropic	1396 (45.3)	743 (62.8)	53 (31.0)	98 (58.3)	92 (48.2)	2427 (49.7)
Antidepressants	1108 (35.9)	584 (49.3)	47 (27.5)	70 (41.7)	71 (37.2)	1919 (39.3)
Antipsychotics^b^	47 (1.5)	15 (1.3)	< 3	22 (13.1)	< 3	87 (1.8)
BZDRs	609 (19.7)	405 (34.2)	18 (10.5)	57 (33.9)	48 (25.1)	1153 (23.6)
Any cardiovascular disorder	2438 (79.1)	833 (70.4)	111 (64.9)	117 (69.6)	144 (75.4)	3693 (75.6)
Diabetes	539 (17.5)	210 (17.7)	17 (9.9)	27 (16.1)	25 (13.1)	831 (17.0)
Gastric acid disorder	1370 (44.1)	596 (50.3)	57 (33.3)	71 (42.3)	86 (45.0)	2219 (45.4)
Reactive airway disease	617 (20.0)	331 (28.0)	22 (12.9)	31 (18.5)	39 (20.4)	1059 (21.7)
Osteoporosis/Paget’s	400 (13.0)	111 (9.4)	9 (5.3)	13 (7.7)	14 (7.3)	551 (11.3)

*DA* = dopamine agonist; *MAO-B* = monoamine oxidase B; *NSAIDs* = nonsteroidal anti-inflammatory drugs; *BZDRs* = Benzodiazepines and related drugs

^a^Characteristics of initiators of ergot DAs (*n* = 30), amantadine (*n* = 42) and LD + COMT (*n* = 17) were not reported separately due to low number of users. However, these were included in the total number of new users of anti-Parkinson medications

^b^Antipsychotic use was measured during the prior year excluding the three months preceding the date of initiation of anti-Parkinson medication

Treatment initiation with levodopa was most frequent overall and ranged from 61 to 65% (shown in Supplementary Fig. 1A, Supplementary Table 2). Initiation of non-ergot DAs increased from 22% in 2013/2014 to 27% in 2016/2017 (RR 1.23, 95% CI 1.02–1.47). The majority of initiations with non-ergot DAs comprised of pramipexole initiations (98.7%) and only 1.4% of persons initiated with rotigotine. Initiation with anticholinergics decreased from 6.9% to 2.4% (RR 0.34, 95% CI 0.21–0.55). Initiation with MAO-B inhibitors or combination therapy ranged between 3–4% whereas initiation with amantadine, ergot-derived DAs, or levodopa with catechol-o-methyltransferase inhibitor (LD + COMT), varied below 1% for each group (shown in Supplementary Fig. 1B, Supplementary Table 2).

**Fig. 2:**
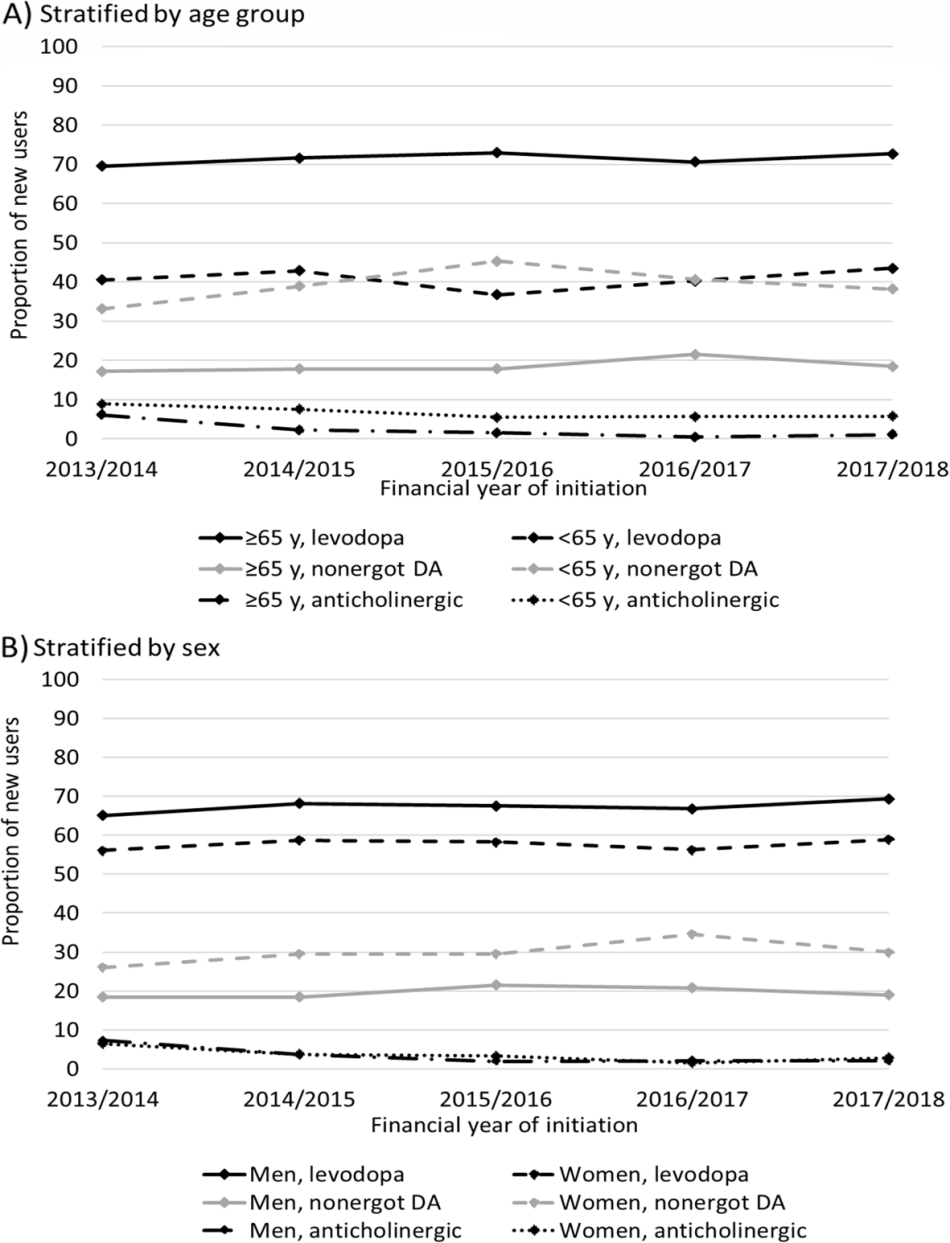
Proportion of new users who initiated with levodopa, non-ergot dopamine agonists (DA), or anticholinergics for each financial year **A**) stratified by age (< 65 years vs. ≥ 65 years); or **B**) stratified by sex

Levodopa was the most commonly initiated medication among persons aged ≥ 65 years, varying from 70 to 73% (shown in Fig. 2A). Initiation with non-ergot DAs was less frequent among older initiators and was highest (22%) in 2016/2017 compared to 2013/2014 (17%). Among younger persons, initiation with non-ergot DAs (45%) exceeded initiation with levodopa (37%) only in 2015/2016 (shown in Fig. 2A)*.* Otherwise, initiation with levodopa was similar or more frequent than initiation with non-ergot DAs. Persons aged < 65 years compared to those aged ≥ 65 years initiated more frequently with anticholinergics (8.9%-5.7% vs. 6.1%-1.1%) throughout the follow-up. Compared with older people, younger people were also more likely to initiate with MAO-B inhibitors and combination therapy than levodopa (Table 2).

**Table 2:** Predictors of the initiation of different anti-Parkinson medications

	Adjusted odds ratios (ORs), 95% Confidence Intervals (CIs)^a,b^			
	Non-ergot DAs (*n* = 1184)	MAO-B inhibitors (*n* = 171)	Anticholinergics (*n* = 168)	Combination of ≥ 2 groups (*n* = 191)
Aged < 65 years	3.64 (3.04–4.37)	1.66 (1.12–2.47)	6.44 (4.40–9.43)	2.15 (1.48–3.13)
Women	1.55 (1.34–1.80)	0.84 (0.60–1.18)	1.14 (0.82–1.58)	1.04 (0.76–1.41)
Concessional beneficiary	0.97 (0.80–1.18)	0.65 (0.44–0.95)	1.77 (1.16–2.70)	0.79 (0.54–1.15)
Use of propranolol	0.51 (0.34–0.75)	2.11 (1.22–3.65)	1.79 (1.00–3.20)	1.08 (0.58–2.01)
Use of any analgesic	1.64 (1.40–1.93)	0.78 (0.56–1.09)	1.13 (0.79–1.61)	0.91 (0.67–1.25)
Use of antidepressants	1.27 (1.09–1.47)	0.84 (0.59–1.21)	0.84 (0.59–1.19)	0.99 (0.72–1.36)
Use of antipsychotics	0.67 (0.36–1.24)	0.49 (0.07–3.62)	8.56 (4.72–15.52)	0.64 (0.15–2.68)
Use of BZDRs	1.70 (1.45–2.01)	0.60 (0.36–1.00)	1.59 (1.10–2.31)	1.40 (0.98–1.99)
Any cardiovascular disorder	0.77 (0.64–0.93)	0.72 (0.50–1.05)	0.87 (0.58–1.30)	1.10 (0.75–1.62)
Diabetes	1.09 (0.90–1.32)	0.69 (0.41–1.16)	1.11 (0.70–1.74)	0.75 (0.48–1.16)
Gastric acid disorder	1.53 (1.29–1.81)	0.80 (0.50–1.27)	1.05 (0.69–1.61)	1.12 (0.77–1.64)
Reactive airway disease	1.26 (1.07–1.47)	1.00 (0.70–1.42)	1.04 (0.72–1.48)	1.21 (0.87–1.67)
Osteoporosis/Paget’s	0.74 (0.58–0.94)	0.57 (0.28–1.15)	0.81 (0.44–1.49)	0.61 (0.35–1.09)

*DA* = dopamine agonist; *MAO-B* = monoamine oxidase B; *BZDRs* = Benzodiazepines and related drugs

^a^Initiators of levodopa (*n* = 3084) were used as the reference

^b^Adjusted for financial year and all variables listed in the table

**Fig. 3:**
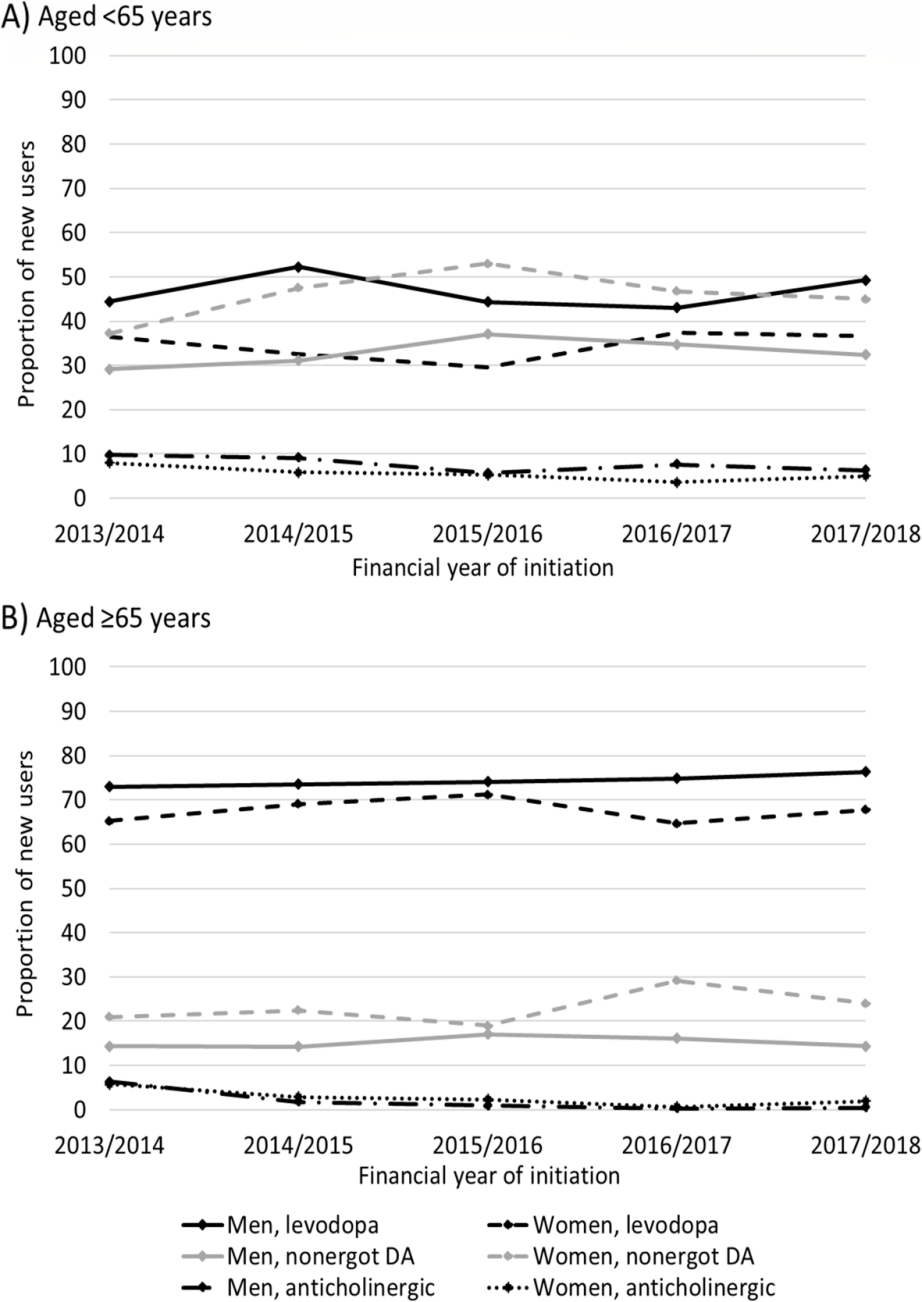
Proportion of new users who initiated with levodopa, non-ergot dopamine agonists (DA), or anticholinergics stratified by sex and age, (**A**) < 65 years; **B**) ≥ 65 years

Women were more likely to initiate with non-ergot DAs (RR 1.46, 95% CI 1.31–1.64 adjusted for financial year and age) and amantadine (2.52, 95% CI 1.31–4.85), and less likely to initiate with levodopa (RR 0.88, 95% CI 0.82–0.94) and MAO-B inhibitors (0.62, 95% CI 0.45–0.85) compared to men (shown in Fig. 2B). Among women aged < 65 years, initiation with DAs (37%) was as frequent as initiation with levodopa (37%) in the first financial year but exceeded initiation with levodopa thereafter (shown in Fig. 3A). Among men aged < 65 years, initiation with levodopa (44%-49%) was more frequent than initiation with non-ergot DAs (29%-32%) throughout the follow-up. In both men and women aged ≥ 65 years, initiation with levodopa was most common but still less frequent among women (65%-68%) than among men (73%-76%) (shown in Fig. 3B). In addition to age and sex, concessional status, use of analgesics, antidepressants, antipsychotics and benzodiazepines and related drugs, and specific comorbidities such as cardiovascular disorders, reactive airway diseases, and gastric acid disorder were associated with initiation of different anti-Parkinson medication (Table 2).

## Discussion

This is the first national study to report the patterns of treatment initiation for PD in Australia. Overall initiation with levodopa remained stable from 2013 to 2018, whereas initiation with non-ergot DAs increased and anticholinergics decreased. Women were more likely to initiate treatment with non-ergot DAs than men. Non-ergot DAs were preferred over levodopa as initial medication in women aged < 65 years after 2014, whereas levodopa was preferred among men aged < 65 and ≥ 65 years throughout the study period.

Our finding that levodopa was preferred as initial therapy among persons aged ≥ 65 years is consistent with guidelines [1–3, 5, 10, 13]. There is no Australian National Health and Medical Research Council guideline for the treatment of PD. However, the widely used Therapeutic Guidelines: Neurology [13] recommend levodopa due to the favourable benefit-to-risk ratio, particularly for persons aged > 70 years or with cognitive impairment. Among persons aged < 65 years, the initial choice varied between levodopa and non-ergot DAs during the five years. Similarly, DAs were preferred as initial medication among persons aged < 60 years in Finland in 2005 and 2012 [17]. Although not directly recommended in the Therapeutic Guidelines [13], initiation with DA in younger persons is consistent with international clinical guidelines [2, 3, 5, 10], with a view to avoiding or delaying levodopa-induced motor complications.

We identified different treatment approaches among men and women despite this not being recommended in guidelines. Levodopa-induced dyskinesias and milder initial symptoms are more likely among women [18, 19], which could explain why DAs were preferred among women. In addition, differences in predominant symptoms, individual life circumstances, preferences, and physician’s attitudes could affect the treatment choice. However, PBS data did not permit analyses of these factors in our study. A recent study from the United States [20] found women had delayed treatment initiation and longer time to treatment switches and add-on than men. Men also use higher levodopa equivalent doses than women but this may reflect dosage adjustment according to patient’s body weight and symptoms [18]. Our findings are similar to an Israeli study [21] that found men aged 75–84 years had higher rate of levodopa initiation than women and younger men aged 50–74 years had higher rates of MAO-B inhibitor use than younger women. Contrary to our findings, older men had higher rates of DA use compared to older women [21]. Further studies are needed to establish the generalizability of our findings to other populations, and to investigate the reasons for and outcomes of preferential initiation with DAs among women.

Treatment initiation with anticholinergics declined in all age groups. This may be because anticholinergics are considered relatively ineffective and associated with anticholinergic adverse events such as confusion, cognitive decline and hallucinations [1, 3, 5] Persons who initiated anticholinergics were more likely to have used propranolol during the preceding year than levodopa initiators, suggesting anticholinergics may have been used for tremor-dominant symptoms. However, apparent initiation with anticholinergics, particularly early in the study period, was unexpected because the Therapeutic Guidelines only recommend anticholinergics as alternatives or adjunct medications after specialist referral if response to levodopa is inadequate [13]. In comparison, treatment was rarely initiated with anticholinergics in Finland in 2012 [17]. Because anticholinergics are mainly used for drug-induced extrapyramidal adverse events [22, 23], we excluded persons who used antipsychotics, prochlorperazine, or metoclopramide in the three months prior to initiation of anti-Parkinson medications. Despite this, initiators of anticholinergics were more likely to have used antipsychotics during the preceding year (13.1%) than initiators of levodopa (1.5%). It is possible, therefore, that our results represent an overestimate of the true rate of treatment initiation with anticholinergics for primary PD because anticholinergic may have been used for the secondary parkinsonism related to antipsychotic use.

Initiation with MAO-B inhibitors was rare throughout the study period. In contrast, initiation with MAO-B inhibitors increased from 18.5% to 33.5% in persons aged < 60 years and from 11.9% to 17.0% in persons aged 60–74 years in Finland from 2005 to 2012 [17]. Several reasons may explain this difference. In Australia, rasagiline was not PBS subsidized for PD for both monotherapy and adjunctive therapy until March 2012 and selegiline is only PBS subsidized for late stage PD as adjunctive therapy [24]. This restriction explains why 91% of MAO-B inhibitor users initiated with rasagiline. The Therapeutic Guidelines state that MAO-B inhibitors have mild effects on symptoms of PD [13]. In comparison, the Finnish Clinical Care Guideline recommends patients aged < 60–65 years and patients with mild symptoms be initiated on either DAs or MAO-B inhibitors [5]. Thus, this difference may reflect the differences in the strength of guideline recommendations and reimbursement practices.

Persons with concessional status were more likely to initiate anticholinergics and less likely to initiate non-ergot DAs and MAO-B inhibitors than levodopa. Anticholinergics were the least expensive anti-Parkinson medications whereas rasagiline was more expensive than most levodopa products. However, the PBS patient co-payment was the same for all medications [25]. Therefore, it is unclear why persons with concessional status were more likely to initiate less expensive medications, unless prescribers were unaware of the cost to the patient. Anecdotally, this might be true for hospital-based prescribers less familiar with Australia’s PBS that predominately reimburses medications dispensed in community and outpatient settings.

### Strengths and limitations

A strength of our study is that the 10% random sample is representative for all 25 million Australian residents. We restricted the study period to July 2013 onwards to capture all the under co-payment dispensings. However, the data did not include records of medications not listed on the PBS. General limitations of dispensing data are that it does not include information on diagnoses, type or severity of symptoms*.* We used anti-Parkinson medication dispensing as a proxy for PD. We cannot be certain that all new users had primary PD rather than other movement disorders. However, we excluded persons who initiated pramipexole products for severe restless legs syndrome, and cabergoline and bromocriptine for hyperprolactinemia. No validation studies have investigated the use of PBS item codes by prescribers. Therefore, it is possible we excluded some persons with primary PD and vice versa. Our results regarding use of anticholinergics and ergot-derived DAs may represent an overestimate.

## Conclusions

Levodopa was the most commonly initiated medication among persons aged ≥ 65 years. However, treatment initiation with DA has become increasingly common relative to levodopa among women but not among men aged < 65 years. Differences in the pattern of treatment initiation among men and women deserve further investigation.

## Supplementary Information

**Additional file 1:** **Supplementary Figure 1** Proportion of new users who initiated with (A) levodopa, non-ergot dopamine agonists, or anticholinergics; or with (B) other anti-Parkinson drugs for each financial year. (DA=dopamine agonist; MAOBI=Monoamine oxidase B inhibitors; LD+COMT=levodopa + catechol-o-methyltransferase inhibitor)

**Additional file 2:** **Supplementary Table 1** Definitions of baseline covariates

**Additional file 3:** **Supplementary Table 2** Proportions and rate ratios of the initiation of different anti-Parkinson medication for each financial year

## Data Availability

The data that support the findings of this study are available from Services Australia. Restrictions apply to the availability of these data, which were used under license for this study.

## Abbreviations

ATC: Anatomical Therapeutic Chemical
CI: Confidence interval
DAs: Dopamine agonists
IQR: Interquartile ranges
LD + COMT: Levodopa plus catechol-o-methyltransferase inhibitor
MAO-B: Monoamine oxidase B
NSAIDs: Nonsteroidal anti-inflammatory drugs
OR: Odds ratio
PBS: Pharmaceutical Benefit Scheme
PD: Parkinson’s disease
RR: Rate ratio
