# Supplementary material for: Treatment initiation for parkinson’s disease in Australia 2013–2018: a nation-wide study
Source: BMC Geriatr. 2022 Jun 3;22:483. doi: 10.1186/s12877-022-03095-3 (PMC9166304; doi:10.1186/s12877-022-03095-3)
Supplement: Supplementary file 1 — Additional file 1: Supplementary Figure 1 Proportion of new users who initiated with (A) levodopa, non-ergot dopamine agonists, or anticholinergics; or with (B) other anti-Parkinson drugs for each financial year. (DA=dopamine agonist; MAOBI=Monoamine oxidase B inhibitors; LD+COMT=levodopa + catechol-o-methyltransferase inhibitor) [file 12877_2022_3095_MOESM1_ESM.docx]

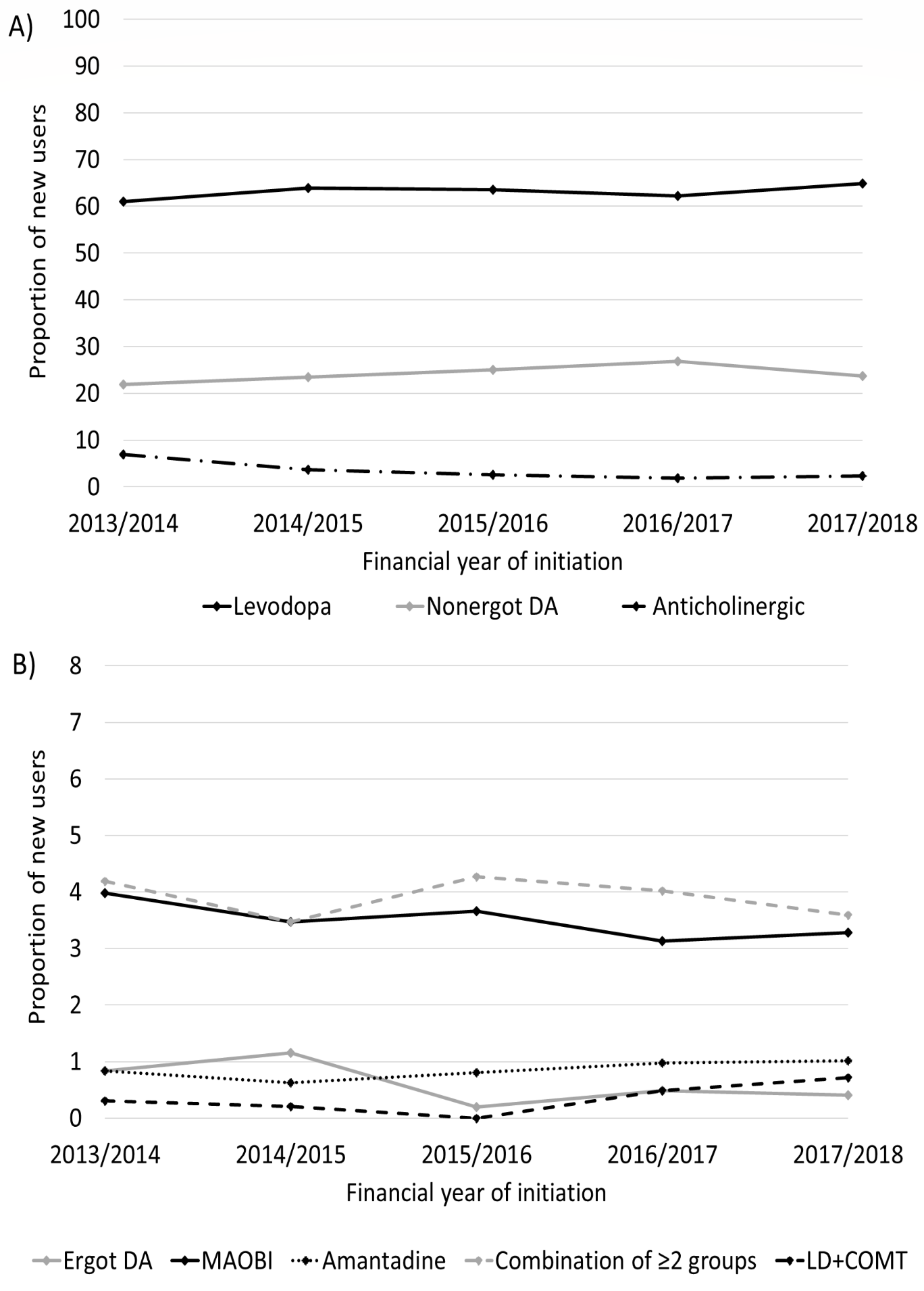


Supplementary Figure 1. Proportion of new users who initiated with A) levodopa, non-ergot dopamine agonists, or anticholinergics; or with B) other anti-Parkinson drugs for each financial year. (DA=dopamine agonist; MAOBI=Monoamine oxidase B inhibitors; LD+COMT=levodopa + catechol-o-methyltransferase inhibitor)
