# Supplementary material for: Treatment initiation for parkinson’s disease in Australia 2013–2018: a nation-wide study
Source: BMC Geriatr. 2022 Jun 3;22:483. doi: 10.1186/s12877-022-03095-3 (PMC9166304; doi:10.1186/s12877-022-03095-3)
Supplement: Supplementary file 2 — Additional file 2: Supplementary Table 1 Definitions of baseline covariates [file 12877_2022_3095_MOESM2_ESM.docx]

Supplementary Table 1. Definitions of baseline covariates

| Covariate | Anatomical Therapeutic Chemical Classification codes^a^ |
| --- | --- |
| Use of propranolol | C07AA05 |
| Use of opioids | N02AA01-N02AX99, R05DA04 |
| Use of NSAIDS | M01AB01-M01AH06 |
| Use of pregabalin | N03AX16 |
| Use of paracetamol | N02BE01 |
| Use of antidepressants | N06AA01-N06AG02, N06AX03-N06AX11, N06AX13-N06AX26 |
| Use of antipsychotics | N05AA01-N05AB02, N05AB06-N05AL07, N05AX01-N05AX17 |
| Use of benzodiazepines and related drugs | N05BA, N05CD, N05CF |
| Any cardiovascular disorder | Anticoagulants B01AA03-B01AB06, B01AE07, B01AF01, B01AF02, B01AX05  Antiplatelets B01AC04-B01AC30  Arrhythmia C01AA05, C01BA01-C01BD01, C07AA07  Congestive heart failure/hypertension C03DA04, C07AB07, C07AG02, C07AB12, C09DX04, C07AB02_2^b^, [(C03CA01-C03CC01) AND (C09AA01-C09AA16, C09CA01-C09CX99)]  Hyperlipidemia C10AA01-C10BX12  Hypertension C03AA01-C03BA11, C03BB04, C03DA01-C03DA03, C03EA01-C03EA14, C09BA02-C09BA15, C09DA01-C09DA09, C02AB01-C02AC05, C02DB01-C02DB04, C03DB01-C03DB02, [(C03CA01-C03CC01) OR (C09AA01-C09AA16, C09CA01-C09CX99)]  Ischemic heart disease/angina C01DA02-C01DA70, C01DX16, C08EX02  Ischemic heart disease/hypertension C07AA01-C07AA06, C07AG01, C08CA01-C08DB01, C09DB01-C09DB08, C09DX01-C09DX03, C09BB02-C09BB12, C07AB03, C07AB02_1^c^ |
| Diabetes | A10AA01-A10BX08 |
| Gastric acid disorder | A02BA01-A02BX77 |
| Reactive airway disease | R03AC02-R03DC03, R03DX05 |
| Osteoporosis/Paget’s | M05BA01-M05BB08, M05BX03, M05BX04, H05AA02 |

^a^Where the ATC code has _01 or_02 at the end, this can be used for multiple indications; therefore, to separate indications based on Pharmaceutical Benefits Scheme item codes, these additional digits were used.

^b^C07AB02_2=metoprolol succinate item codes that have restricted benefit for moderate to severe heart failure

^c^C07AB02_1=metoprolol tartrate item codes
