# Supplementary material for: Treatment initiation for parkinson’s disease in Australia 2013–2018: a nation-wide study
Source: BMC Geriatr. 2022 Jun 3;22:483. doi: 10.1186/s12877-022-03095-3 (PMC9166304; doi:10.1186/s12877-022-03095-3)
Supplement: Supplementary file 3 — Additional file 3: Supplementary Table 2 Proportions and rate ratios of the initiation of different anti-Parkinson medication for each financial year [file 12877_2022_3095_MOESM3_ESM.docx]

Supplementary Table 2. Proportions and rate ratios of the initiation of different anti-Parkinson medication for each financial year

|  | 2013/2014^a^ | 2014/2015 | | 2015/2016 | | 2016/2017 | | 2017/2018 | |
| --- | --- | --- | --- | --- | --- | --- | --- | --- | --- |
| Initial medication | % | % | RR (95% CI) | % | RR (95% CI) | % | RR (95% CI) | % | RR (95% CI) |
| Levodopa | 61.0 | 64.0 | 1.05 (0.94-1.17) | 63.5 | 1.04 (0.93-1.17) | 62.2 | 1.02 (0.91-1.14) | 64.9 | 1.06 (0.95-1.19) |
| Non-ergot DA | 21.9 | 23.4 | 1.07 (0.89-1.29) | 25.0 | 1.14 (0.95-1.37) | 26.8 | 1.23 (1.02-1.47) | 23.8 | 1.09 (0.90-1.31) |
| Ergot DA | 0.8 | 1.2 | 1.38 (0.55-3.43) | 0.2 | 0.24 (0.05-1.14) | 0.5 | 0.58 (0.19-1.79) | 0.4 | 0.49 (0.15-1.62) |
| MAO-B inhibitor | 4.0 | 3.5 | 0.75 (0.26-2.17) | 3.7 | 0.97 (0.36-2.58) | 3.1 | 1.17 (0.46-2.96) | 3.3 | 1.22 (0.48-3.10) |
| Anticholinergic | 6.9 | 3.7 | 0.53 (0.35-0.80) | 2.5 | 0.37 (0.23-0.58) | 1.9 | 0.27 (0.16-0.45) | 2.4 | 0.34 (0.21-0.55) |
| Amantadine | 0.8 | 0.6 | 0.87 (0.55-1.39) | 0.8 | 0.92 (0.58-1.45) | 1.0 | 0.79 (0.49-1.26) | 1.0 | 0.82 (0.51-1.32) |
| LD+COMT | 0.3 | 0.2 | 0.67 (0.11-4.00) | 0.0 | NA | 0.5 | 1.56 (0.37-6.52) | 0.7 | 2.28 (0.59-8.82) |
| Combination of ≥2 groups | 4.2 | 3.5 | 0.83 (0.52-1.31) | 4.3 | 1.02 (0.66-1.57) | 4.0 | 0.96 (0.62-1.48) | 3.6 | 0.86 (0.54-1.35) |

DA=dopamine agonist; MAO-B=monoamine oxidase B; LD+COMT=levodopa + catechol-o-methyltransferase inhibitor

^a^Financial year 2013/2014 was used as a reference category
